# “*I’d keep going until somebody said no and nobody ever said no*”: exploring identity-strengths amongst medical students from widening participation backgrounds

**DOI:** 10.3389/fmed.2025.1530738

**Published:** 2025-07-14

**Authors:** Kathrine Gibson Smith, Jennifer A. Cleland, Kim Walker, Colin Lumsden, Anita Laidlaw

**Affiliations:** ^1^School of Medicine, Medical Sciences and Nutrition, University of Aberdeen, Aberdeen, United Kingdom; ^2^Lee Kong Chian School of Medicine, Nanyang Technological University, Singapore, Singapore

**Keywords:** widening participation, widening access, strengths based approach, anti-deficit, medicine

## Abstract

**Background:**

Widening participation is an important consideration in medicine, which has been historically elitist. Despite the evolving evidence base on WP in medicine and advancing of discourse around conceptualizing WP, the identity of students from WP backgrounds remains problematized. Currently, there are few studies exploring how WP medical students have found strength in their experiences of adversity and developed skills which will be an asset to their career. To address this gap, the aim of this study is to understand, using a strengths-based approach, the strengths and resources that WP students draw on to enact success in medicine.

**Methods:**

This is a qualitative study using individual interviews for data collection. We selected the identity-specific strength lens to understand how WP students in medicine draw on their strengths and resources to enact success in pursuing careers in medicine. We recruited eligible students who had completed, or were undertaking, a ‘gateway’ programme and had directly entered the undergraduate medical degree and who had fulfilled university WP criteria prior to entry. Interviews were recorded electronically and transcribed verbatim. Data were coded inductively and in accordance with thematic analysis.

**Results:**

Five main themes were constructed from the analysis. Participants drew on the following strengths and resources to enact success in medicine: (i) Not taking no for an answer: determination and perseverance; (ii) Learning from the past: using their lived experience; (iii) Making things work: resourcefulness; (iv) Drawing on their sense of self: Self-awareness, reflection and independence; and, (v) Growing a support network: Having strong relationships with others.

**Conclusion:**

There is still much to be done in creating inclusive environments in medical education which promote belonging and development of diverse values and beliefs. However, using strengths-based approaches can reframe study of widening participation in medicine and shift thinking and discourses from deficit to anti-deficit ways of thinking and discourses.

## Background

Governments across the globe have developed and adopted policy to widen participation to higher education. The ultimate aim being to promote participation in higher education amongst groups who are typically underrepresented (1, 2). Enactment of widening participation (WP) policies varies according to the context and history of marginalization [e.g., in the United Kingdom (UK) the focus tends to center on social mobility, whereas in Australia, the prevailing discourse is in relation to social accountability (2, 3)]. There is also considerable variation in terms of the demographic groups which WP seeks to target. For example, even within the UK, there are significant differences across academic institutions in terms of who qualifies as WP (1).

Widening participation is an important consideration in medicine, which has been historically elitist (3). Recent evidence suggests that, still, the largest number of learners in medicine have been educated at more privileged schools (4, 5). Despite evidence of persistent privilege in medicine, there is increasing diversity in learners within medical education partly due to the proliferation of Initiatives which have been embedded across medical schools globally to increase representation of individuals traditionally underrepresented within the medical profession (6, 7). With this, there is a growing evidence base which has enhanced understanding and knowledge in relation to WP in medicine, and which has illustrated the continued structural and systemic inequalities that WP learners experience across the continuum of medical education (6, 8, 9). Despite the evolving evidence base on WP in medicine and advancing of discourse around conceptualizing WP, the identity of students from WP backgrounds remains problematized. What we mean by this is that an individual’s identity and background are framed as potential barriers to success (7, 10). For example, attributing the prevalence of certain personality traits as individual deficiencies (11). Such understanding is termed as a ‘deficit’ way of conceiving WP e.g., WP as a problem to be solved (11, 12).

Burke et al. (13) argue that the adoption of this deficit model inflicts further harm to students from underrepresented backgrounds since it fails to recognize, and further develop, the values that diverse student learners bring to educational institutions. Accordingly, adoption of a deficit-based lens to detangle the experiences of those who have been historically underrepresented in medicine obfuscates the depth of experience that individuals have outside the realm of systemic inequality (e.g., persistent privilege), and which can positively impact on their, and others, learning (14). Anti-deficit, or strengths-based, approaches pose an alternative lens to position WP learners and offer a means to explore how institutional cultures and traditions are not configured in a way which promote belonging amongst diverse groups (12). Accordingly, integration of anti-deficit approaches within research in HPE would step away from problematizing WP (e.g., framing of aspects of an individual’s background and identity as barriers to success) and seek to embed perspectives of flourishing, or thriving, into ways of thinking (11, 15).

Yet this is not straightforward. Research and interpretations using strength frameworks need to avoid the trap of focusing on the need for the learner his or herself to enact change (16), rather than focusing change efforts at structural and systemic barriers. Similarly, there needs to be due consideration given to avoid replacing negative stereotypes with positive counters, as these also perpetuating troubling discourses and limit opportunity for the dismantling of systemic inequality, in relation to privilege, for underrepresented groups (17). For example, the term grit, often considered as a facet of resilience, can be positioned within a deficit approach (e.g., placing the onus on the learner to develop grit), or, more positively, within an anti-deficit lens (e.g., how grit may be developed amongst learners as they transcend systemic inequalities) (11).

Currently, there are few studies exploring how WP medical students have found strength in their experiences of adversity and developed skills which will be an asset to their career (8). There is limited understanding of how such students may have transformed their experiences to enact success [e.g., an individual’s ability to not just “get in” but “get on” (7, 10)] in an environment where they continue to face structural and systemic inequalities. To address this gap, the aim of this study is to understand, using a strengths-based approach, the strengths and resources that WP students draw on to enact success in medicine. By doing so, we hope to move away from problematizing WP in medicine and toward developing an understanding of how WP students thrive in an unequal system.

## Materials and methods

### Conceptual framework

Of the various strengths-based approaches outlined within the literature [e.g. (18)], we selected the identity-specific strengths lens owed to its relevance, and previous use in higher education (14), to inform our way of thinking and conceiving how WP students in medicine draw on their strengths and resources to enact success in pursuing careers in medicine. The identity-specific strengths approach, also termed background-strengths (14), postulates that individuals who have experienced marginalization often acquire important skills, knowledge and perspectives as a consequence of their backgrounds and associated experiences (18). In the context of education, the identity-strengths, such as an ability to persist in the face of challenges, of WP students can be valuable resources beneficial to the individual’s education and to society more generally (18). Hence, the framework fits neatly with both notions of social justice, including social mobility, in WP to medicine and WP as a means to potentially enhance patient care via the proposition of ‘like treat like’ (6).

### Design

The work is concerned with individuals’ experiences and interpretations of their own realities within a social context and is therein positioned in relation to an interpretivist worldview within a social constructivist paradigm (19). This is a qualitative study using individual interviews for data collection.

### Context

The research was conducted in Scotland, UK. There is a significant policy focus in Scotland which has been driven by the Scottish Government in collaboration with the wider higher education sector on widening access to higher education (20, 21). There are several policies and programmes which have been implemented in an effort to reduce the gap in educational attainment which has been evident across socioeconomic groups. These have included free tuition for Scottish-domiciled students, contextual admissions, ring-fenced university places, scholarships and outreach programmes (20). In this context, WP initiatives are focused on supporting those from less traditional/privileged backgrounds to pursue a career in medicine e.g., potential students who have resided in one of Scotland’s 20% most deprived areas (22), are care experienced, attended a school with lower progression rates to higher education (for example, in a remote and rural area), etc. This study was conducted at a Scottish University where there are two entry routes into medicine for students from WP backgrounds: via a ‘gateway’ programme; and, via standard entry into medicine.

The ‘gateway’ programme was established by the University in 2017 in response to a Scottish Government call (21) to further support entry for students from target areas into medicine. Students on the ‘gateway’ programme are required to fulfill predetermined academic performance criteria in their ‘gateway’ year and undertake an admissions test (23) and a Multiple Mini Interview (24) to confer entry to first year of medicine within the institution.

Students from WP backgrounds also enter medicine via the standard entry route (e.g., directly to Year 1 of the medical programme). Contextualized (holistic) admissions (25) may be applied via standard entry routes and consider aspects of an applicant’s background [including home postcode, whether they are care-experienced, are a young carer or registered for the Reach National Schools Programme (26)] when making offers to get into medicine (e.g., reducing the required grades for entry).

### Participants and recruitment

Eligible students were those who had completed, or were undertaking, the ‘gateway’ programme and those who had directly entered the undergraduate medical degree and who had fulfilled university WP criteria prior to entry (as outlined above). Class email lists of students who were currently enrolled in the ‘gateway’ programme were passed to the lead researcher (KGS). Students enrolled in the undergraduate medical degree programme were also recruited via class email lists which were sent out by year administrators. A recruitment email, containing an information sheet, was sent by KGS or a year administrator. Two reminder emails were sent. Participants were asked to express their interest via email and a consent form was sent. Students were recruited from all years across the ‘gateway’ programme and undergraduate medical degree.

### Data collection

Data collection was undertaken by KGS, who has experience conducting qualitative interviews, over a 3-year period (2021–2024) either in-person in a small teaching room on university premises or virtually via Microsoft Teams. Students were captured at various points over the course of their studies – from ‘gateway’ to year five of medicine – and one student was interviewed twice.

Interviews were informed by a semi-structured interview guide which had been developed by the research team with reference to the wider literature on WP to medicine. This guide was used flexibly in accordance with participants’ responses. It covered aspects of participant’s experiences of the ‘gateway’ programme, where relevant, and transitions into and across their medical degree programme. Questions took an anti-deficit approach and incorporated a style of questioning similar to those outlined in the anti-deficit achievement framework (15). The framework outlines ‘instead of’ ways of thinking to counter common deficit ways of thinking which means looking at how success is enacted in the face of challenges. We encouraged students to reflect on how they had succeeded in the face of adversity and were continuing to thrive, as well as exploring how they negotiated entry into medicine and made connections. All interviews were digitally recorded on an audio recorder and were uploaded to a secure university shared drive prior to transcription by an approved transcriber and anonymized. The interview audio was then deleted on the recording device.

We used the concept of information power to guide our sample size (27) in that we (i) required a medium-sized sample since our aim was relatively broad, (ii) were recruiting specific participant characteristics, (iii) intended to utilize theory to interpret findings, (iv) attained a strong dialogue with participants, and (v) used a cross-case approach to analysis. We continually and iteratively appraised the data collected, to check for data sufficiency. We stopped data collection after 25 interviews.

Participant demographic information is not presented to ensure anonymity although WP status was framed as having met at least one of the following criteria: SIMD20 postcode; young person who was care experienced (e.g., looked after by the local authority in care or care leaver); first generation applicant to higher education; eligible for Educational Maintenance Award; evidence of severe financial hardship not reflected by SIMD categorization; not schooled in English prior to secondary school; completed a Gateway programme (for which there were additional entry criteria, including: SIMD40; estranged; eligible for free school meals; resident in remote and rural area as per Scottish Government Urban Remote Classification; eligible refugee or asylum seeker). However, we can say that all twenty-five participants fulfilled WP criteria, 17 students were from the Gateway programme and 8 were direct entry. Of those who elected to disclose, two-thirds of students had resided in an area with a SIMD20 postcode (22). The data captured experiences across the Gateway programme (*n* = 6) and all years of the medical degree (Yr1 = 3, Yr2 = 3, Yr3 = 4, Yr4 = 4, Yr5 = 5).

### Data management and analysis

The data in the transcripts were coded inductively (e.g., codes were developed from the data), in accordance with a thematic analysis as per Braun and Clarke (28), and therein analysed formally both manually, using visual mapping techniques, and Microsoft Excel. During this process, KGS identified relevant codes in relation to the research aims and generated themes to form a preliminary analytical framework. Themes were generated from the initial codes and sub-themes, and driven out of the strengths and resources that students felt they had drawn upon to successfully navigate their journey through medicine. Codes were discussed and reviewed by KGS and AL prior to consulting the wider research team for feedback. A revised analytical framework was agreed and applied by KGS and AL, independently, to the transcripts. KGS and AL met to discuss the coding and confirm themes – disagreements were resolved via critical discussion. The coding process was subject to regular review both by KGS and AL, and within the wider research team to enhance trustworthiness (29).

### Ethical approvals

The study received ethical approval from [university ethics committee name] [reference: CERB/2017/8/1501].

## Results

The main findings are presented below in relation to the strengths and resources that the participants had drawn on to enact success in medicine. Five main themes were constructed.

i Not taking no for an answer: determination and perseverance

Participants documented their journeys into and through medicine. They had typically faced challenges navigating the application process with some experiencing a lack of support from their school and family to apply to medicine. They may have had to reapply several times to succeed or consider alternative pathways in (e.g., graduate entry, pre-medicine programmes).

Evident across the students interviewed was their determination and perseverance in the face of these challenges. Although support to apply to medicine was perhaps not in abundance, students were not dissuaded, nor were they deterred from having to consider longer routes into medicine since they were absolutely convinced that medicine was the career for them, “*I’d keep going until somebody said no and nobody ever said no*.”

Participants often discussed how they felt determined to leave their local community as they felt constrained in their life and career choices in these communities. They perceived that schools did not promote progression to higher education, “*I got the distinct impression that provided you didn’t stab someone, get caught selling drugs or get someone pregnant, they didn’t really care what you did post school*,” let alone medicine, and instead encouraged students to go into local rural industries such as farming and fishing. Some described how they longed and aspired for something bigger and greater, and wanted more for themselves than the opportunities they were facing if they stayed within the rural community, “*A lot of people on the island just tend to leave school with little to nothing*…*that’s just how life’s seen and almost like that’s what you do*.”

Participants were determined to succeed – in their studies and in their career aspirations. They were realistic about setbacks, both actual (e.g., having to resit exams) and potential (e.g., in relation to their family circumstances), and were determined about overcoming these. Some felt that although they knew their personal circumstances could pose challenges, they were not simply a product of this. They had high levels of self-confidence and did not view these as integral to their identity, rather they perceived these as challenges that could be overcome, “*I’m not going to let my circumstances, or some exams determine and define who I am because I know I can achieve this and things*.”

Although the students interviewed had successfully got into medicine, they found that establishing a sense of belonging could be difficult, “*I do feel like a fish out of water in a lot of circumstances because I can’t relate to a lot of people*.” They highlighted the hidden curriculum, “*I was concerned about that because again going from the old photographs of medical professors and seeing university and medicine as an institution I was concerned about that. I was concerned about not having a place, not fitting in, being quite different*.” They discussed how traditional university, and professional, practices proved unfamiliar, and they considered, contributed to challenges in establishing a sense of belonging, “*I was with an anesthetist and he’s from [place] as well. He’s “Oh, what school did you go to?” I was like, “[place]” He was like, “Oh, I’m so sorry for you” and it was quite funny*…*it definitely made me feel a lot more out of place*.”. However, although participants spoke to these challenges, they described how they were aware that they brought something to the table that perhaps others could not. Indeed, some students used this as motivation to drive their success and whilst we must be cautious as this speaks to the systemic inequality experienced by students as they enter the higher education institution, it was evident that participants used this to empower themselves, “*I think there’s certainly a type of resilience to be able to face all that and still be like this is what I want to do. I probably use the word spite against people who would detract from me. I need to prove you wrong.*”.

ii) Learning from the past: using their lived experience

Participants were keen to express that whilst they had perhaps experienced hardship, or at least seen it within others in their community or family, they felt they could turn these experiences into strengths in terms of both their outlook on life and also, the life skills they had developed as a consequence. For example, some felt that their knowledge and experience of living in less privileged areas was an asset in terms of being aware of the challenges which patients may face, “…*if you’ve been working and you’re a single parent for a long time, that it’s a lot easier to just bang something in the oven.*”.

This was perceived to be advantageous as they felt that other students, who were from more privileged backgrounds, may not be aware of these experiences. This awareness led to enhanced confidence in terms of speaking to patients since they felt they could offer relatability based on their background and also may have the gift of insight that others do not have in terms of what might matter to the patient, “…*say, if I come across a patient who is also a person of color, they tend to feel more safe and more like they can rely on you and because you understand some of the difficulties that you face as the barriers being a person of color*.”

They felt that their experiences and background meant that they were well suited to overcoming inequalities that were prevalent in healthcare, partly because they were easily able to empathize, “*I think a lot of the problems we face these days can be traced to a sort of generation or two of just middle-class sort of haughty taughty white men basically who think they know everything*.”

We found evidence that participants were determined to pay forward their experiences to those from similar backgrounds and give back to their community in some way. They were often passionate about inspiring other students with backgrounds similar to theirs and were involved in activities which gave something back such as outreach, mentoring, tutoring or going back to their school to talk about their experiences. Much of this was driven by passion to give back and provide opportunities to others that they did not have, “*Like I will happily help them because I wish I had somebody to help me*.”

iii) Making things work: resourcefulness

Access to resources have been viewed as being a key factor in promoting student success (30). However, we found that whilst it is not simply the mere availability of resources that is important, the students in our study discussed how they took ownership over their access to resources and actively negotiated this – some of which were not readily accessible to them and which they needed to negotiate access to. Participants were acutely aware that they needed to effectively utilize and act upon resources to make strides in medicine – both in social and material terms.

They were aware of lacking social capital and confidence compared to their more privileged peers, “…*a few of the others, again a bit, from a bit more middle class or upper-class perspective, they’re a lot more comfortable with just going out there and talking to whoever they want to, whatever specialist they want to.*”. However, whilst students described feeling on the back foot to their colleagues in terms of the creation of professional connections, they effectively utilized resources to their benefit and made active effort to grow their network in medicine – often using placement opportunities to develop a network, seeking out assistance from academic staff tutors to leverage opportunity or via their peers who they perceived to be more privileged, “*And just kind of started asking, well if other students are doing it, how are they getting research opportunities*.”

Participants were also proactive in utilizing university support resources from those directed toward pastoral care and wellbeing to developing academic skills. Ultimately, they were confident in knowing themselves and what they needed. Some students felt they lacked the academic preparedness, owed to having been educated in a school with lower progression rates to university, that they perceived others had. However, often through a process of trial and error, and at times failure, they figured out ways of learning that worked for them. They sought help from peers and dedicated support staff to advance their knowledge and recognized when they were struggling, “… *in first and second year, I wasn’t studying the right way, I was like studying the way I did at school and then in third and definitely in fourth year I got a different way of studying for I was working on my memory.*”.

The link between financial struggle and WP permeated most of the interviews within this study. Many participants were balancing part time work with their studies and described the priority paid work had to take since it funded their living costs. It meant they sometimes had to forgo opportunities such as engaging in research, extracurriculars, studying or attending university for lectures. Students however, described the lengths they went to, to make things work – to balance their time, to stretch their finances and take care of themselves. Some had learned from their previous experiences, parents had shown how they could manage finances and maintain their wellbeing, *“I’m generally really good with money because my mum raised me on such a strict budget*… *the woman’s a mastermind with money.*”.

iv) Drawing on their sense of self: Self-awareness, reflection and independence

Participants talked about experiences of adversity (e.g., parental estrangement, caring for family members with addiction/mental health issues, having been care-experienced) and trauma (e.g., personal mental health struggles, experiences of homelessness). They were keen to take ownership of their own narratives and share their stories.

Some described post-traumatic growth [e.g., how positive change had occurred after exposure to trauma (31)] and positive adaptation as a consequence of the adversity they had experienced, “…*this sounds really grim, and when I say, but I actually have to say I’m kind of, I’m happy that it’s been like that [experience of caring for family member with mental health problem] because it’s shown me how to bounce back and it’s shown me resilience*.” Others described how they used negative experiences as a form of empowerment. For example, one student, who had experienced racial microaggressions felt that they could use these experiences to empower themselves in the future, “*I think I’d say even facing racism and microaggressions*… *even though they’re negative things, they can just give you a kind of a, more of a confidence and it allows you to be able to stand up for yourself*.”

Students often reflected on their personal growth and development of independence, particularly when considering context, and took great pride in what they achieved, “*I’m proud to say that this is what I’m studying*… *even though I’ve had to come via a back passage into it, I’ve still made it there*.” They also expressed that they felt gratitude for they believed that their life could have taken an alternate direction and were keen to dispel any notion that they were a victim – rather they felt, they were a master of their own destiny and not a passive recipient of what they had, “*I feel like thinking like that or letting those things get to me makes me feel as if I’m thinking of myself as a victim.*”.

Students found ways to manage their own health and wellbeing where there were challenges and also, that of those they cared for, “*My mum’s quite unwell which has always been a difficult thing to manage and cope with studies*…*I’m very good at compartmentalizing like uni and personal life*.”. They were adaptable, knew how to self-care and were aware of their mental wellbeing knowing what they needed to do for themselves to maintain positive wellbeing whilst achieving their goals, “*I need to overwork myself to make sure I pass my exams, but I’m also trying to lay myself back*…*I want to come out and still love the career that I’ve wanted. I’m hoping to just keep the passion all the way through*.”

v) Growing a support network: Having strong relationships with others

The positive role of developing positive relationships with others, including family, peers, academic or clinical staff, was highlighted in this study. Participants talked about being able to draw on trusted relationships to develop their confidence, promote positive wellbeing and develop their social, and professional networks further. Being socially connected was an asset. For example, one student discussed how social networks served multiple purposes and they recognized how they were beneficial to maintaining a positive outlook and promoting personal growth “*I think the social networks are, one it just helps you get out of your comfort shell and then, two, as well, just being able to bounce ideas off each other and if you’re low, speaking to each other and just being able to increase morale between one another*.”.

Their relationships allowed them to grow and develop as a young adult and begin their professional identity development. Academic staff and clinical tutors were valuable in developing participants’ confidence, particularly so where individuals no longer had contact with their parents or family. Trusted relationships with members of staff provided a pastoral and confidence boosting role in their lives, “… *all I really needed, I think, was someone to believe that I could and also give me a bit of a kick up the arse and sort of go, “Right, come on, you can get As and you can do this*.”

## Discussion

The aim of this study is to understand, using a strengths-based approach, the strengths and resources that WP students draw on to enact success in medicine, and by doing so move away from problematizing WP in medicine and toward developing an understanding of how WP students thrive in an unequal system where persistent privilege is still at play. Our findings highlighted that participants navigate inequality throughout their journey of trying to gain a place in medical school and their time at medical school by drawing on identity strengths and resources (18). These identity-strengths included determination and perseverance; resourcefulness; using their lived experience; developing self-awareness, reflection and independence; and establishing strong social networks. Students were not only cognizant of the identity resources they needed to draw on to succeed but also knew, or developed an understanding, of how they could operationalize these to leverage their career aspirations. In line with the identity-strength based approach (18), background and lived experience appeared to be pivotal in empowering students and providing them with confidence.

Whilst they often discussed feeling different to others in medicine, owed to their background (e.g., rural or lower socioeconomic status), participants simultaneously felt that their life experiences allowed them to better relate to patients [see also (32, 33)]. They drew strength from this and challenged deficit views in relation to their identity (18). Participants also demonstrated a clear sense of self – they were self-aware, knew their strengths, were able to self-reflect on their journey at key points of their educational experience to better themselves, and they also demonstrated how they had developed independence and used this to build confidence.

As in previous research (1), participants were often balancing part time work with their studies – they noted this came at a significant cost to their ability to engage with the course both academically and in extracurricular activities. However, they discussed how having limited financial resource meant they felt better able to manage their finances. There was also clear evidence that for some rural students, they were determined to make something different of their lives having felt they were constrained by expectations that they would stay local. This echoes research on rural student negotiation of identity and place, where the intersection between rurality and socioeconomic privilege was highlighted. This speaks to the importance of considering, where appropriate and possible, the various intersections of WP (33).

Lastly, they knew the value of having strong social ties and developing a social network which they could use for both personal support but also which they could draw on to develop themselves professionally. These findings echo previous research (34) wherein students from WP backgrounds in medicine recognized and mobilized weak social ties to create linking capital although encountered difficulties, once in medicine, in creating social ties.

Our findings illustrate a story of positivity – students from WP backgrounds have many identity strengths to bring to the table. However, we need to be cautious in interpreting whether students from WP backgrounds have more or different identity strengths than their more privileged peers. Similarly, we must also reflect on whether the level or extent of identity resources needed to succeed in medicine for WP students is different from that of their peers. Our findings highlight that systemic privilege is still at play, despite large scale global investment in increasing diversity in the medical profession and reinforce the need to change the discourse about WP to medicine.

Accordingly, it is perhaps useful to consider how our findings in relation to individual identity strengths fall into place within the wider system of medical education, and how they may be cultivated to counter persistent privilege. We can draw on Bronfenbrenner’s (35) Ecological Systems Theory (EST) of human development to further understand how various subsystems could interact within the medical education context to enable WP students to thrive in medicine. EST posits five ecological systems and suggest that proximal processes (bidirectional interactions) exist between an individual and their environment (35). The five systems proposed by Bronfenbrenner include the: microsystem (e.g., immediate contexts in addition to the people in these contexts who interact with the individual); mesosystem [e.g., influences between actors of microsystems (for example, university and family relationships)]; exosystem [e.g., external influences unrelated or departed from the microsystem (for example, educational and government policy)]; macrosystem (e.g., cultural and social influences such as socioeconomic status); chronosystem (e.g., changes across systems such as life transitions) (36, 37). EST helps us to frame the outcome of success of WP as a product of interaction between the individual and the systems they find themselves within – critically, this removes the onus being on the learner and their inherent traits and highlights the role that the environment has on influencing individual success.

Although we recognize that the framing of WP students’ personal qualities as strengths may not be entirely novel in approach, our findings illustrate how WP students have thrived and flourished despite the systemic and structural inequalities they have faced in higher education institutions and in medicine. These illustrations are representative of an effort to shift the discourse of WP in medicine from problematizing learners toward understanding the strengths that can be brought to medical careers from students from diverse backgrounds. If WP learners can thrive in environments where equity is lacking, how might they flourish if the educational and medical milieu truly embraced, and supported, diverse learners? What could students achieve if we were to eradicate the systemic inequalities inherent within these systems? Importantly, we further question, why is the academic bar for medicine set so high whilst significant other ‘achievements’ such as those identified in this study are unrecognized currently within admissions systems? Hence our findings are not only relevant to WP learners in healthcare professions education but the wider field of WP in higher education.

Given that retention of doctors in medical practice in the UK is a current issue threatening the sustainability of the workforce (38), it would perhaps be prudent to consider individual strengths and how they might feed into long term resilience of the workforce. This fits with Cleland et al.’s (39) guide to robust, defensible and fair selection into medical school wherein they outline how when considering admissions into medicine is critical to consider the goals of the selection process e.g., the type of doctors we need to graduate. Accordingly, perhaps as highlighted by our evidence, there should be greater emphasis at the point of selection on the individual strengths applicants bring to a career in medicine such as perseverance, resourcefulness and self-awareness – with consideration given to these in relation to other contextual factors e.g., socioeconomic status, care-experienced etc.

We made efforts to recruit a wide and representative group of participants. They came from diverse WP backgrounds - lower SES, care leavers, etc., - and there was breadth in terms of what they had experienced. However, they also identified challenges that were specific to subpopulations within the WP category (for example, care-leavers). Whilst we recognize the importance of conducting analyses via an intersectional lens, we were constrained within this study due to the population size at the recruitment site and duty to protect participant confidentiality. There remains much work to be done in developing an understanding on intersectionality within WP students (8). The study was carried out in one place and so the findings may not be transferable to other contexts. However, the WP criteria locally reflect those in many other countries (2, 40, 41) and our use of identity-specific strengths (18) as a theoretical framework will aid conceptual generalizability. The study design did not allow us to assess how students develop and draw on identity-strengths throughout their educational journey. We suggest that these come into play early in life rather than developing at the point of considering medical school. This needs further investigation: longitudinal qualitative work (42) and/or narrative accounts of students’ stories, for example using a life story interview (43), are needed.

In conclusion, there is still much to be done in creating inclusive environments in medical education which promote belonging and development of diverse values and beliefs. However, using strengths-based approaches can reframe study of widening participation in medicine and shift thinking and discourses from deficit to anti-deficit ways of thinking and discourses.

## Data Availability

The datasets presented in this article are not readily available because participants could be identified by data the information in the data.
